# Depression is associated with heart failure in patients with type 2 diabetes mellitus

**DOI:** 10.3389/fpubh.2023.1181336

**Published:** 2023-05-25

**Authors:** Yanying Chen, Chen Long, Zhenhua Xing

**Affiliations:** ^1^Department of Cardiovascular Medicine, Second Xiangya Hospital, Central South University, Changsha, Hunan, China; ^2^Department of General Surgery, Second Xiangya Hospital, Central South University, Changsha, China; ^3^Department of Emergency Medicine, Second Xiangya Hospital, Central South University, Changsha, China; ^4^Trauma Center, Second Xiangya Hospital, Central South University, Changsha, China; ^5^Emergency Medicine and Difficult Diseases Institute, Second Xiangya Hospital, Central South University, Changsha, China

**Keywords:** heart failure (HF), 9-item Patient Health Questionnaire (PHQ-9), type 2 diabetes mellitus (T2DM), depression, mental health

## Abstract

**Background:**

Type 2 diabetes mellitus (T2DM) is associated with an increased risk of heart failure (HF). Depression, a common comorbidity of T2DM, may further increase the risk of heart failure (HF). We investigated the association between depression and incident HF in patients with T2DM.

**Methods and results:**

Depressive symptoms were assessed in the ACCORD Health-Related Quality of Life study participants at baseline, 12, 36, and 48 months using the nine-item Patient Health Questionnaire (PHQ-9). The severity of depressive symptoms was categorized as none (0–4 points), mild (5–9 points), or moderate-severe (10–24 points). Cox regression with PHQ-9 as a time-dependent covariate was used to assess the association between depression and incident HF. During the median follow-up of 8.1 years, 104 participants developed HF (incidence: 7.1/1,000 person-years). Half of the participants with moderate-severe depression were relieved and a significant percentage of participants without depression or with mild depression worsened to mild or moderate-severe depression during the follow-up period, respectively. Each unit increase in the PHQ-9 score was associated with a 5% higher risk of HF (hazard ratio [HR]:1.05, 95% confidence interval [CI]: 1.01–1.10). Patients with depression ever (HR: 2.23, 95% CI: 1.25–3.98) or persistent depression (HR: 2.13, 95% CI: 1.05–4.44) had a higher risk of HF than those without depression ever.

**Conclusion:**

Depressive symptoms change greatly in T2DM patients, depressive symptoms are an independent risk factor for HF. These results reinforce the importance of continuous evaluation and management of mental health status in T2DM patients with high HF risk.

## Introduction

Type 2 diabetes mellitus (T2DM) has become an emerging epidemic and a major clinical and public health concern (1). Poor mental health is an additional concern in patients with T2DM. Approximately one in every four patients with T2DM suffers from clinically significant depression (2). T2DM increases the risk of depression, and depression increases the risk of hyperglycemia and insulin resistance, which in turn worsen T2DM.

Patients with T2DM have a higher risk of developing cardiovascular disease (CVD) than those without (3). The risk of heart failure (HF) is significantly considerably higher and the prevalence of HF in patients with T2DM is up to four times higher than that in the general population (4, 5). Depression is also one of the most prevalent symptoms in patients with HF. Symptoms of depression worsen the quality of life and the prognosis of patients with established HF (6, 7). However, it is unclear whether depression is a risk factor for HF or an incidental comorbidity of HF, due to different populations and instruments used to assess depressive symptoms (8–12). None of the previous studies have investigated the specific effects of depression on HF incidence among patients with T2DM who have a higher risk of both depression and HF than those without T2DM. Previous studies have not considered dynamic changes in depressive symptoms during the follow-up period. Persistent and transient depression may have distinct effects on the incidence of HF.

The Action to Control Cardiovascular Risk in Diabetes (ACCORD) trial provided a unique opportunity to investigate the relationship between depression and HF in T2DM patients. In this study, we aimed to investigate the prospective association of dynamic depressive symptoms with the risk of subsequent HF among patients with T2DM, taking into account traditional CVD risk factors.

## Methods

### Study population and data collection

The rationale, design, and primary outcomes of the ACCORD study have been described and published previously (13, 14). Briefly, the ACCORD study, including 10,251 patients (mean age 62 years and mean glycated hemoglobin [HbA1c] 8.3%) with a median onset of T2DM 10 years previously, was designed to assess whether intensified control over blood glucose, blood pressure, and lipid levels could improve CVD outcomes. After an average follow-up of 3.7 years, the intervention was discontinued because intensive glycemic control increased the risk of cardiac death, and all participants transitioned to standard glycemic control and follow-up was continued.

The ACCORD Health-Related Quality of Life (HRQL) study was a substudy of the ACCORD study, which was designed to prospectively assess the overall effect of intensive intervention on validated measures of depression and HRQL from the participant's point of view. Depressive symptoms were assessed in all ACCORD HRQL sub-study participants using the nine-item Patient Health Questionnaire (PHQ-9), which is based on the Diagnostic and Statistical Manual of Mental Disorders (DSM-IV) criteria, at baseline and at 12, 36, and 48 months during ACCORD study clinical visits. The PHQ-9 included nine questions, each of which was graded on a scale of 0 to 3 based on the severity of the symptom. The severity of depressive symptoms was categorized as none (0–4 points), mild (5–9 points), or moderate-severe (10–24 points). Participants with a history of HF at enrollment were excluded from the analysis. A flowchart of the study is shown in Supplementary Figure 1. The ACCORD trial was approved by an NHLBI review panel and the ethics committee at each center.

### Covariates

The baseline characteristics of the participants, including age, sex, race (white/non-white), duration of T2DM (years), living alone history of CVD or HF (with/without), proteinuria (with/without), family history of CVD, tobacco and alcohol consumption, and medications, were obtained using questionnaires, interviews, and medical records at recruitment. Smoking status was categorized as never, former, or current. Alcohol consumption was self-reported and was measured as times per week. Body mass index (BMI), systolic blood pressure (SBP), diastolic blood pressure (DBP), and heart rate (HR) were measured by registered nurses at the assessment center. Lipids (total cholesterol, triglycerides, low-density lipoprotein cholesterol [LDL], and high-density lipoprotein cholesterol [HDL]), HbA1c, and glomerular filtration rate (GFR) were measured by the central laboratory, as described previously (5, 13, 15, 16).

The outcome of interest in our study was the incidence of HF during the follow-up period, defined as the first hospitalization for HF or death due to congestive HF. Hospitalizations due to HF were assessed based on clinical and radiological evidence. Death due to HF without clinical or postmortem evidence of an acute ischemic event was defined as death due to HF. A central committee adjudicated the HF events according to a predefined protocol (5, 13, 14). Time to event was calculated as the number of years until the occurrence of an HF event. Participants were censored at the time of their last follow-up.

### Statistical analysis

Continuous variables were compared using analysis of variance or Mann–Whitney U tests, and categorical variables were compared using chi-square analysis according to the distribution type. Cox proportional hazards regression with PHQ-9 as a time-dependent covariate was used to assess the association between depression and HF incidence. We analyzed the associations using these two models. Model 1 was adjusted for age, race, sex, glucose control strategy, CVD history, living alone, educational status, and cigarette and alcohol consumption. Model 2 was further adjusted for BMI, total cholesterol, triglycerides, LDL, HDL, SBP, DBP, HR, and GFR, in addition to the adjustment variables in model 1. We further adjusted for family history of CVD, and medications, including antidepressant drugs and beta-blockers, to test whether the association between depression and HF incidence was affected by family history of CVD and medication use. The medications were also treated as time-dependent covariates and synchronized with the PHQ-9 test. Subgroup and interaction analyses were performed according to age ( ≤ 60 years, >60 years), sex, glycemic control strategy (intensive or standard glucose control), and CVD history. All statistical analyses were two-sided, and *P* < 0.05 were considered statistically significant. All analyses were performed using Stata/MP software, version 17.0 (StataCorp LLC, College Station, TX, USA).

## Results

**Figure 1:**
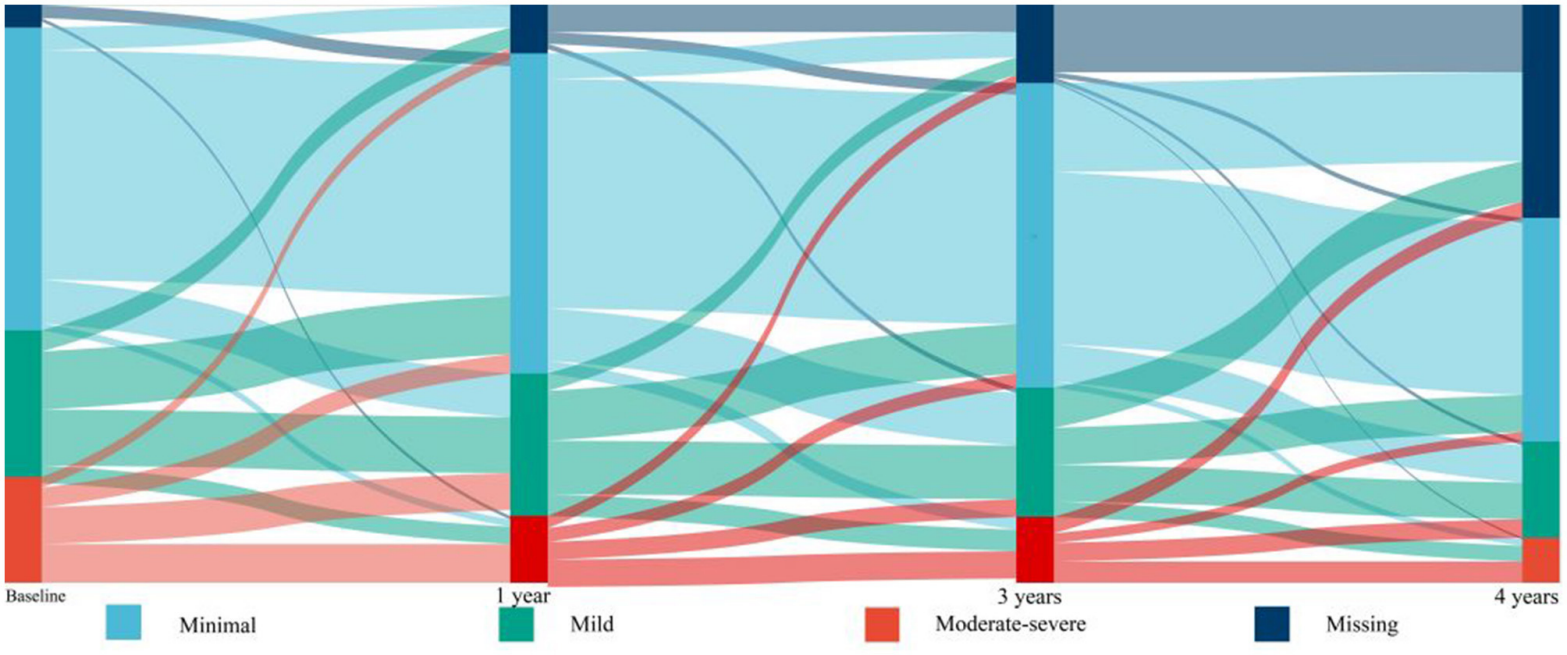
Depressive symptoms at baseline and 12, 36, and 48 months showing changes over time.

Of the 10,251 participants included in the ACCORD study, 2,053 participated in the HRQL sub-study. One hundred participants without baseline PHQ-9 data and 100 participants with a history of HF were excluded from the study. A total of 1,853 participants were included in the analysis. During a median follow-up period of 8.1 years (interquartile range: 6.1–10.1 years), 104 participants developed HF (7.1 events per 1,000 person-years). The baseline characteristics of study participants are shown in Table 1. Participants with HF were more likely to be older, have a longer history of T2DM, higher HbA1c levels, and lower DBP than those without HF. Female participants and participants with a history of CVD were more likely to develop HF than male participants without a history of CVD. Table 2 shows the prevalence and incidence of mild or moderate-severe depression based on the PHQ-9 score at baseline and during follow-up (Table 2). A total of 489 participants (26.4%) had mild depression and 354 participants (19.1%) had moderate-severe depression at baseline. Figure 1 shows the dynamic changes in depressive symptoms during the follow-up period. The prevalence of moderate-severe depression decreased during the follow-up period. Depressive symptoms changed during the follow-up period: half of the participants with moderate-severe depression experienced relief of their symptoms, and a substantial percentage of participants without depression or with mild depression developed mild or moderate-severe depression, respectively, but participants without depression at baseline were unlikely to develop moderate-severe depression. There are an increased proportion of data of PHQ-9 categorized as “missing”, 5.4% at baseline, 8.9% at 1 year, 14.6% at 3 years, and 37.5% at 4 years, respectively.

**Table 1:** Baseline characteristics of participants based on heart failure status.

	**Heart failure status**		***P*** **-value**
**N**	104 (Yes)	1749 (No)	
**Age (years)**	64.68 ± 7.56	62.60 ± 6.46	0.002
**Sex, male (%)**	24 (23.08%)	718 (41.05%)	< 0.001
**Race, White (%)**	69 (66.35%)	1111 (63.52%)	0.561
**Glycemia control (%)**			0.871
Standard	53 (50.96%)	877 (50.14%)	
Intensive	51 (49.04%)	872 (49.86%)	
**Duration of diabetes (years)**	13.08 ± 8.82	10.87 ± 7.59	0.005
**CVD history (%)**	65 (62.50%)	555 (31.73%)	< 0.001
**Live alone (%)**	13 (12.50%)	354 (20.24%)	0.054
**Education (%)**			0.075
Less than high school	23 (22.12%)	238 (13.62%)	
High-school graduate	28 (26.92%)	450 (25.76%)	
Some college	31 (29.81%)	575 (32.91%)	
College degree or higher	22 (21.15%)	484 (27.70%)	
**Proteinuria**	18 (17.31%)	306 (17.50%)	0.961
**Current Smoker**	13 (12.50%)	228 (13.04%)	0.875
**Drinking/week**	0.79 ± 2.29	0.95 ± 2.66	0.545
**BMI (Kg/m** ^**2**^ **)**	32.90 ± 5.31	32.34 ± 5.38	0.305
**Lipid (mg/dl)**			
CHOL	181.05 ± 41.89	183.40 ± 41.01	0.572
TRIG	198.00 ± 133.57	188.12 ± 141.27	0.489
LDL	102.10 ± 31.08	104.64 ± 33.88	0.457
HDL	40.41 ± 11.67	42.41 ± 11.58	0.088
**HBA1C (%)**	8.49 ± 1.02	8.25 ± 1.04	0.025
**SBP (mmHg)**	138.49 ± 16.13	136.42 ± 16.89	0.223
**DBP (mmHg)**	71.90 ± 9.61	74.82 ± 10.79	0.007
**HR (beats/min)**	70.52 ± 11.62	72.71 ± 11.85	0.068
**GFR (ml/min/1.73m** ^**2**^ **)**	91.52 ± 24.06	92.39 ± 31.83	0.785
**Medication**			
ARB	17 (16.35%)	312 (17.85%)	0.697
ACEI	57 (54.81%)	914 (52.29%)	0.617
β-blocker	51 (49.04%)	505 (28.89%)	< 0.001
Sulphonylurea	52 (50.00%)	954 (54.55%)	0.366
Metformin	70 (67.31%)	1132 (64.72%)	0.592
Meglitinide	0 (0.00%)	39 (2.23%)	0.124
Thiazolidinedione	22 (21.15%)	415 (23.73%)	0.548
Insulin	20 (19.23%)	186 (10.63%)	0.007
Statin	70 (67.31%)	1108 (63.50%)	0.432
Aspirin	70 (67.31%)	969 (55.53%)	0.019
Anti-depression drug	13 (12.50%)	241 (13.81%)	0.706

CVD, cardiovascular disease; LDL, low-density lipoprotein cholesterol; HDL, high-density lipoprotein cholesterol; SBP, systolic blood pressure; DBP, diastolic blood pressure; HR, heart rate; GFR, Glomerular filtration rate.

**Table 2:** Participants meeting depression criteria at assessment points through ACCORD trial.

**Visit**	**N**	**No depression (PHQ-9 0-4)**	**Mild (PHQ-9 5-9)**	**Moderate-severe (PHQ-9 10-27)**
Baseline	1,853	1,010 (54.5%)	489 (26.4%)	354 (19.1%)
1 year	1,767	1,068 (60.5%)	476 (26.9%)	223 (12.6%)
3 years	1,668	1,016 (60.9%)	431 (25.9%)	221 (13.2%)
4 years	1,218	746 (61.2%)	322 (26.5%)	150 (12.3%)
Ever	–	1,501	1,044	577

Table 3 shows the association between the PHQ-9, both as a continuous variable and a categorical variable, and HF. Using model 2, each unit increase in the PHQ-9 score was associated with a 5% increase in the risk of HF. Patients with mild depression had higher risk of HF (hazard ratio [HR]: 2.26, 95% confidence interval [CI]: 1.38–3.69) than those without depression. Although participants with moderate-severe depression also had a higher risk of HF (HR: 1.47, 95% CI: 0.72–2.99) than those without depression, but the increased risk was not statistically significant because of the limited number of participants with moderate-severe depression. Using model 2, both patients with depression ever (HR: 2.23, 95% CI: 1.25–3.98), and patients with persistent depression (HR: 2.13, 95% CI: 1.05–4.44), had higher risk of HF than those without depression ever.

**Table 3:** Proportional hazard models of depression predicting heart failure.

	**HR (95%CI)**		
	**Model 1**	**Model 2**	**Model 2**+**medication and family history of CVD**
None	Ref	Ref	Ref
Mide	2.30 (1.42, 3.73)	2.26 (1.38, 3.69)	2.25 (1.35, 3.71)
Moderate-severe	1.65 (0.82, 3.33)	1.47 (0.72, 2.99)	1.52 (0.73, 3.14)
P for trend	0.01	0.03	0.03
PHQ9 continuous	1.06 (1.01, 1.11)	1.05 (1.01, 1.10)	1.05 (1.01, 1.10)
Ref	Ref		Ref
Ever depression (*N* = 1,571)	2.43 (1.37, 4.31)	2.23 (1.25, 3.98)	2.11 (1.16, 3.75)
Persistent depression (*N* = 479)	2.54 (1.25, 5.18)	2.13 (1.05, 4.44)	2.19 (1.11, 4.51)

Model 1: age, race, sex, glucose control strategy, CVD history, living along, education status, cigarette and alcohol consumption.

Model 2: age, race, sex, glucose control strategy, CVD history, living along, education status, cigarette and alcohol consumption, BMI, Total cholesterol, triglyceride, LDL, HDL, SBP, DBP, HR, GFR.

Medication including ARB, ACEI, β-blocker, Sulphonylurea, Metformin, Meglitinide, Thiazolidinedione, Insulin, Statin, Aspirin, Anti-depression drug.

CVD, cardiovascular disease; LDL, low-density lipoprotein cholesterol; HDL, high-density lipoprotein cholesterol; SBP, systolic blood pressure; DBP, diastolic blood pressure; HR, heart rate; GFR, Glomerular filtration rate.

Subgroup and interactive analyses were performed to test the robustness of the association between the PHQ-9 and HF risk. Age (≤60 years or >60 years), sex, glycemic control strategy (intensive or standard glucose control), and CVD history did not play an interactive role in the associations between PHQ-9 and incidence of HF (Supplementary Figure 2). When further adjusted for family history of CVD and medications, including antidepressants and beta-blockers, in addition to model 2, the results were robust and unchanged (Table 3).

## Discussion

This *post-hoc* analysis of the ACCORD HRQL study showed that depressive symptoms changed dynamically during the follow-up period, and that in patients with T2DM, depression at baseline or during follow-up period were associated with a higher risk of HF. This risk remained generally unchanged even after adjustment for demographic characteristics, CVD risk factors, and medication use, including antidepressant drugs. This finding indicates that it is important to identify depression in patients with T2DM because they are at higher risk of developing HF.

Previous studies on depression and risk of HF have been conducted in non-diabetic populations and have had conflicting results (8–12). A *post-hoc* analysis of the HUNT study cohort revealed that symptoms of depression at baseline were associated with an increased risk of HF in a dose-response manner (12). However, another *post-hoc* analysis of the Established Population for Epidemiologic Studies of the Elderly (EPESE) cohort did not find such an association (8). There are several possible reasons for these conflicting results. First, several different depression questionnaires, with different diagnostic performance, were used by these studies. For example, the Center for Epidemiological Studies Depression Scale used in the EPESE study has not been validated for widespread use (17). Second, the depressive symptoms changed markedly during the follow-up period. Previous studies have only included symptoms at baseline without re-evaluation during follow-up periods of more than 10 years (8–12). Participants without depression at baseline but with depression during the follow-up period may have a higher HF risk than those without depression during the follow-up period.

In contrast to previous studies, all participants in the ACCORD study had T2DM with CVD or a higher risk of CVD; thus, they had a higher risk of HF than previous studies (6, 18). Therefore, our study might have had more power to detect an association between depressive symptoms and incidence of HF than previous studies. Previous studies ignored the dynamic changes in depressive symptoms during the follow-up period. Some patients with moderate depression experience recurrence, whereas others do not (19). Our present study found that a large proportion of patients with depression experienced relief. Patients without depression develop moderate-to-severe depression. This study found that participants without depression at baseline but with depression during the follow-up period had a comparable risk of HF as those with depression at baseline.

Our study has several limitations. First, as in previous studies, our predefined outcomes did not distinguish between HF with reduced ejection fraction and HF with preserved ejection fraction. Second, the sample size was relatively small. Due to the small number of patients with moderate-severe depression, we did not detect significantly higher risk of moderate-severe depression and CI became wide. Furthermore, we did not detect whether antidepressant drugs could reduce the risk of HF incidence due to limited sample size. Third, the use of drugs that may be effective for HF, such as SGLT2 inhibitors and GLP-1 receptor agonists, and the use of such drugs was underrepresented in the study cohort because recruitment to the ACCORD study ended in 2005. Fourth, all the participants were from North America, and these findings may not apply to other populations with different characteristics and lifestyles.

## Conclusion

Depression is an independent risk factor for HF and depressive symptoms change dynamically in patients with T2DM. These results reinforce the importance of continuous evaluation and management of mental health in patients with T2DM and a high risk of HF.

## Data availability statement

The original contributions presented in the study are included in the article/Supplementary material, further inquiries can be directed to the corresponding author.

## Ethics statement

The ACCORD trial was approved by an NHLBI Review Panel and the Ethics Committee at each center. The patients/participants provided their written informed consent to participate in this study. Ethical review and approval was not required for the animal study because none.

## Author contributions

ZX designed the study and provided methodological expertise. YC and CL drafted the manuscript. All authors have read, provided critical feedback on, and approved the final manuscript.
