# Supplementary material for: Depression is associated with heart failure in patients with type 2 diabetes mellitus
Source: Front Public Health. 2023 May 25;11:1181336. doi: 10.3389/fpubh.2023.1181336 (PMC10248230; doi:10.3389/fpubh.2023.1181336)
Supplement: Supplementary file 1 [file Table_1.DOCX]

# Depression is associated with heart failure among type 2 diabetes mellitus

Yanying Chen, MD^1^ ; Chen Long, MD^2^; Zhenhua Xing, MD^3,4,5*^

1. Department of Cardiovascular Medicine, The Second Xiangya Hospital, Central South University, Changsha, Hunan, China.

2.Department of General Surgery, The Second Xiangya Hospital, Central South University, Changsha, China.

3. Department of Emergency Medicine, Second Xiangya Hospital, Central South University, Changsha 410011, China

4. Trauma center, Hunan province; Trauma center, Second Xiangya Hospital, Central South University, Changsha 410011, China;

5. Emergency Medicine and Difficult Diseases Institute, Second Xiangya Hospital, Central South University, Changsha 410011, China.

*Corresponding author: Zhenhua Xing, MD

E-mail: xing2012x@csu.edu.cn

Phone number: +8615084714930

Total word count: 2500

Trial registration: http://www.clinicaltrials.gov. Unique identifier: NCT00000620

**Supplementary figure 1: flow chart of present study**


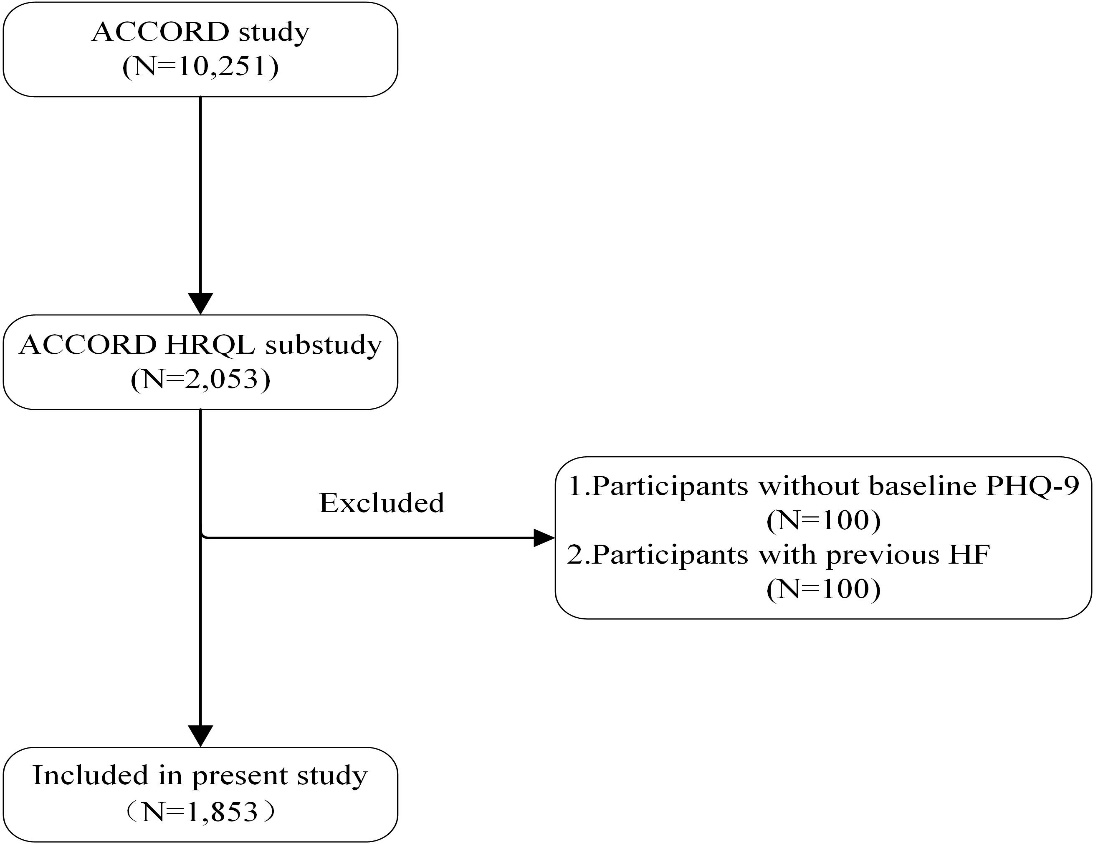


**Supplementary Figure 2, Subgroup and interactive analysis.**


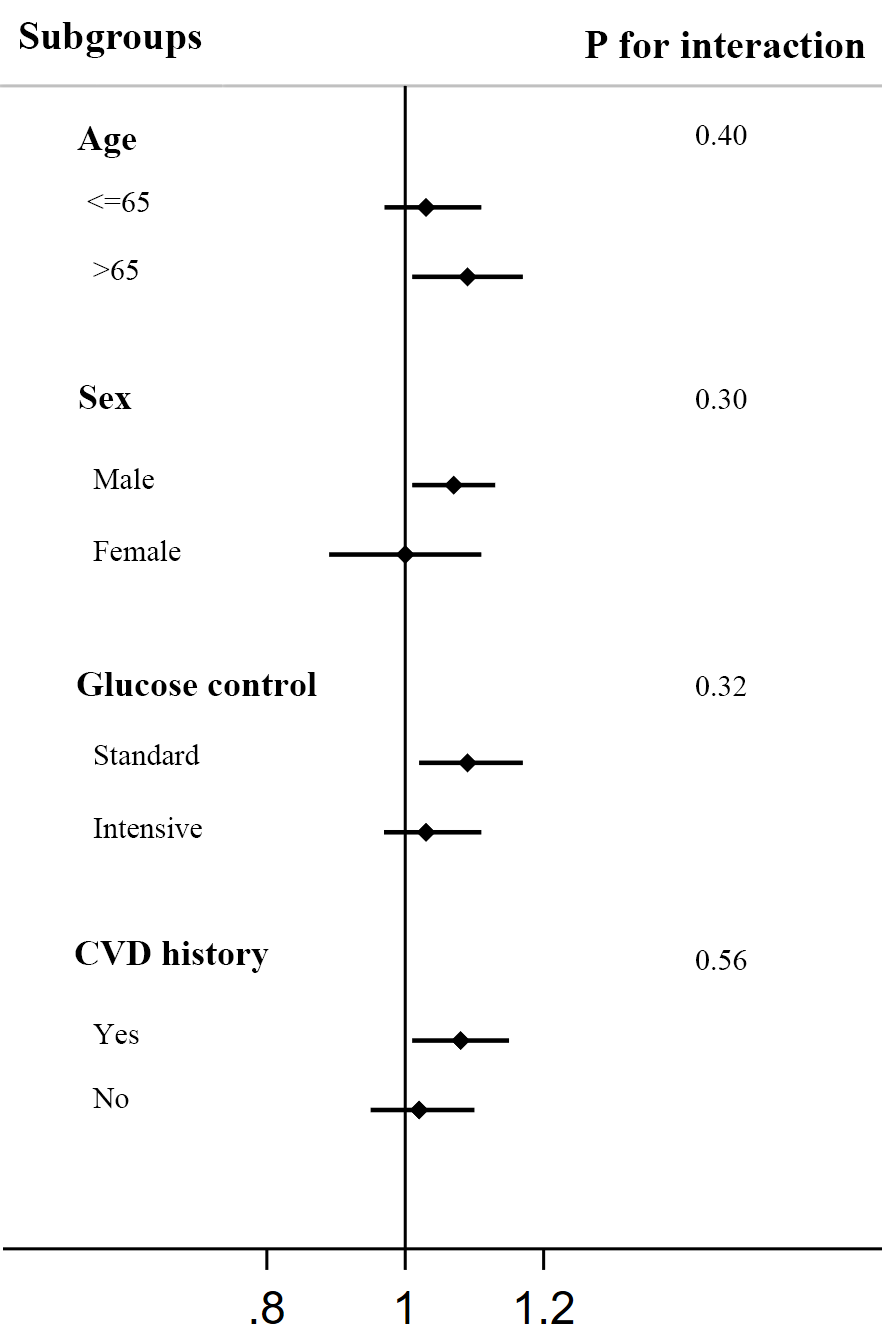


Hazard ratios per 1 standard deviation increase in PHQ-9 for the HF. Each stratification was adjusted for all factors in model 2, except for the stratification factor itself.
